# The Role of APOE4 in Disrupting the Homeostatic Functions of Astrocytes and Microglia in Aging and Alzheimer’s Disease

**DOI:** 10.3389/fnagi.2019.00014

**Published:** 2019-02-11

**Authors:** Celia G. Fernandez, Mary E. Hamby, Morgan L. McReynolds, William J. Ray

**Affiliations:** The Neurodegeneration Consortium, Institute of Applied Cancer Science (IACS), The University of Texas MD Anderson Cancer Center, Houston, TX, United States

**Keywords:** APOE, Alzheimer’s disease, astrocytes, microglia, aging

## Abstract

APOE4 is the greatest genetic risk factor for late-onset Alzheimer’s disease (AD), increasing the risk of developing the disease by 3-fold in the 14% of the population that are carriers. Despite 25 years of research, the exact mechanisms underlying how APOE4 contributes to AD pathogenesis remain incompletely defined. APOE in the brain is primarily expressed by astrocytes and microglia, cell types that are now widely appreciated to play key roles in the pathogenesis of AD; thus, a picture is emerging wherein APOE4 disrupts normal glial cell biology, intersecting with changes that occur during normal aging to ultimately cause neurodegeneration and cognitive dysfunction. This review article will summarize how APOE4 alters specific pathways in astrocytes and microglia in the context of AD and the aging brain. APOE itself, as a secreted lipoprotein without enzymatic activity, may prove challenging to directly target therapeutically in the classical sense. Therefore, a deeper understanding of the underlying pathways responsible for APOE4 toxicity is needed so that more tractable pathways and drug targets can be identified to reduce APOE4-mediated disease risk.

## Introduction

Alzheimer’s disease (AD) is a devastating neurodegenerative disease of aging, the incidence of which is expected to increase exponentially as the proportion of the population over the age of 65 increases. Research in AD drug discovery has historically focused on the Amyloid Hypothesis, based primarily on findings from early-onset AD, which is caused by mutations in amyloid-β (Aβ) pathway proteins and which accounts for <2% of all AD cases. While the Amyloid Hypothesis predicts that enhanced production and diminished clearance of Aβ causes AD, therapeutics aimed at modulating Aβ levels have largely failed, although they have not yet been tested at presymptomatic stages of disease (Doig et al., 2017).

After aging, the ε4 allele of the *APOE* gene is the next greatest risk factor for AD, while the relatively rare ε2 allele confers AD protection (Corder et al., 1993; Saunders et al., 1993; Strittmatter et al., 1993). Although 25 years have passed since it was identified, there are still no approved drugs directly targeting APOE4, due partly to the inherent “undruggability” of lipoproteins. However, the atherosclerosis field has demonstrated that indirectly modulating the effect of lipoproteins can be a successful alternative strategy. For example, statins affect lipoprotein composition and disease risk by targeting a metabolic pathway (cholesterol synthesis); similarly, understanding the downstream pathways that mediate APOE4 disease risk might identify more tractable therapeutic targets for treating APOE4-mediated AD.

APOE in the brain is primarily expressed by astrocytes and microglia, and APOE4 expression alters the normal function of both of these glial cell types, potentially contributing to AD risk. Although the toxicity associated with APOE4 likely involves the impaired ability of APOE4-expressing glia to efficiently clear Aβ, it is also apparent that there are Aβ-independent effects on normal glial physiology. The role of APOE in mediating Aβ levels has been discussed in depth elsewhere (Ries and Sastre, 2016), and will only be briefly touched upon below. This review will instead focus on more recent findings that specifically describe the role of APOE in glial biology, in addition to and independent of Aβ modulation, particularly during aging, and will describe pathways in each glial cell type that may link APOE to disease pathogenesis.

Although astrocytes and microglia are the primary producers of APOE, whether an interaction between these cells exists in terms of APOE biology has not been carefully examined. Cross-talk between astrocytes and microglia in neurodegeneration is well-known (Jha et al., 2018); for example, astrocytes can secrete complement factor C3 in response to Aβ, which can then activate microglia *via* the C3a receptor (Lian et al., 2016). On the other hand, lipopolysaccharide-stimulated microglia can induce neurotoxic “A1” reactive astrocytes, as opposed to neurotrophic “A2” reactive astrocytes (Liddelow et al., 2017). The same group found that A1-type astrocytes are present in aging (Clarke et al., 2018) and AD brain (Liddelow et al., 2017), and that A1 astrocytes not only lose the neurotrophic capacity of A2 astrocytes, but also actively produce a neurotoxin to kill neurons and oligodendrocytes. Importantly, a recent study demonstrated that blocking this microglial-dependent induction of A1 astrocytes is protective in mouse models of Parkinson’s disease (Yun et al., 2018). Whether blockade of such microglia/astrocyte cross-talk can help ameliorate neurodegeneration in humans and in AD has yet to be demonstrated. Furthermore, whether APOE is one such secreted factor that mediates interactions between astrocytes and microglia has not been reported, nor has a synergistic effect of APOE from each cell type been clearly defined. Even so, since both astrocytes and microglia express APOE, this review article will separately consider specific aspects of each cell type’s normal physiology that might be impacted by APOE4 expression in aging and AD.

## Overview of APOE Isoforms

APOE is a lipoprotein that normally facilitates lipid transport between cells (Mahley, 1988). *APOE* transcription is activated by liver X receptor (LXR) and peroxisome proliferator-activated receptor γ (PPARγ), transcription factors that regulate lipid homeostasis and inflammation (Laffitte et al., 2001; Akiyama et al., 2002; Liang et al., 2004; Mandrekar-Colucci et al., 2012; Moutinho et al., 2019). In the lipid-rich brain, APOE is predominantly expressed by astrocytes and microglia, and perhaps in limited circumstances by neurons (Boyles et al., 1985; Pitas et al., 1987; Uchihara et al., 1995; Nakai et al., 1996; Xu et al., 1998, 2006).

The human *APOE* gene exists as three different alleles, ε2, ε3, and ε4, which are present at ~7%, 79%, and ~14%, respectively, in the entire population (Bertram et al., 2007), and which exhibit differences in lipid and receptor binding efficiency. The presence of one ε4 allele increases the risk of AD by threefold, while carriers with two ε4 alleles are eight times as likely to develop AD compared to those without any ε4 allele; and ε4 is associated with an earlier age of disease onset, from about 85 years without any ε4, to 75 years with one and 68 years with two ε4 alleles (Corder et al., 1993). These statistics make *APOE* ε4 the greatest known genetic risk factor for AD, more than any other gene to date. In contrast to the human gene, mouse *ApoE* exists as only one isoform, and the structure of the mouse APOE protein more closely matches human APOE3 (Raffai et al., 2001); targeted-replacement mice, in which the endogenous mouse *ApoE* gene has been replaced with either of the human *APOE* isoforms, have therefore been created to study differences in human APOE isoform function, and will be referred to throughout this review article (Sullivan et al., 1997; Knouff et al., 1999).

**Figure 1:**
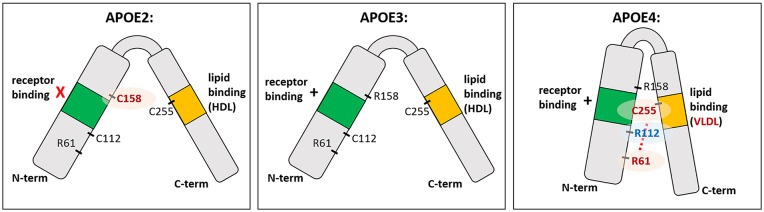
The structure of APOE isoforms. APOE is a soluble secreted protein, with N-terminal and C-terminal domains linked by a central hinge region. The N-terminal domain contains the receptor binding domain (indicated in green), and the C-terminal domain contains the lipid binding region (indicated in orange). Each isoform differs from one another at amino acid position 112 and 158. Cysteine at position 158 (C158) in APOE2 is thought to cause deficient receptor binding, while arginine at position 112 (R112) in APOE4 changes the conformation of the entire domain such that R61 is exposed and interacts with C255 in the C-terminal domain (red dotted line). This “domain interaction” is thought to be the biophysical basis for differences in APOE4 function compared to the other isoforms; e.g., preference for VLDL over HDL. In APOE3 and APOE2, which have C112 instead of R112, the R61 is not exposed and there is no such domain interaction.

The three human APOE isoforms differ from one another in the protein sequence at amino acid positions 112 and 158 (Figure 1; Mahley, 1988; Raffai et al., 2001; Hatters et al., 2006). These single amino acid differences are enough to change the lipid and receptor binding ability of APOE (Weisgraber et al., 1982; Dong and Weisgraber, 1996; Gong et al., 2002). Specifically, R112 in APOE4 creates a domain interaction between the N-terminal receptor binding domain and the C-terminal lipid binding domain, preventing efficient binding to HDL compared to APOE2 and APOE3, with preferential binding to VLDL (Dong et al., 1994; Dong and Weisgraber, 1996). While APOE2 is protective against AD, the ε2 allele is also associated with hyperlipoproteinemia III, which is characterized by accumulated lipoproteins in the plasma and development of atherosclerosis (Giau et al., 2015). This is thought to be caused by impairment in the receptor binding region of APOE2, leading to delayed lipoprotein clearance and increased triglyceride and cholesterol levels (Havel and Kane, 1973; Weisgraber et al., 1982; Mahley and Rall, 2000). In the context of AD, APOE2 has been relatively understudied, although some research is ongoing (Wu and Zhao, 2016). It should be noted that APOE2 in many experimental settings is similar to APOE3 or performs qualitatively better (such as in amyloid clearance). For clarity, and because there is much less in the literature to explain the mechanism of action of APOE2, the present review will focus on different phenotypes conferred by APOE3 vs. APOE4.

### APOE Isoforms and Amyloid Clearance

Both astrocytes and microglia clear Aβ (Paresce et al., 1996; Wyss-Coray et al., 2003; Ries and Sastre, 2016) and although there is some evidence that APOE4 may enhance Aβ production (Ye et al., 2005), it is widely thought that APOE4 confers AD risk through deficient Aβ clearance compared to APOE3 and APOE2 (Koistinaho et al., 2004; Deane et al., 2008; Simonovitch et al., 2016), although not necessarily *via* direct binding (Verghese et al., 2013). APOE isoforms differ not only in lipid binding ability, but also in affinity for specific APOE receptors (Ruiz et al., 2005; Holtzman et al., 2012). LRP1, a major receptor for APOE, mediates Aβ clearance in astrocytes and pericytes (Liu et al., 2017a; Ma et al., 2018), and astrocytes expressing APOE4 have reduced LRP1 surface expression, which could explain impaired amyloid clearance *in vivo* (Prasad and Rao, 2018). However, astrocytes also utilize other APOE receptors such as LDLR for Aβ clearance, but in an APOE-independent manner (Basak et al., 2012); furthermore, Aβ is cleared by transcytosis across the blood brain barrier, glymphatic and interstitial fluid bulk flow, and by extracellular degrading enzymes, highlighting the complexity around understanding how APOE4 contributes to amyloid accumulation.

### APOE Isoforms and Tau Pathology

In addition to modulating Aβ, APOE also affects tau pathology, another hallmark of AD, in an isoform-specific manner. APOE4 worsens tau pathology in the P301S tau mouse model, and APOE4 genotype is associated with exacerbated neurodegeneration in human primary tauopathies (Shi et al., 2017). APOE4 status is associated with tau pathology particularly in instances when amyloid pathology is also present (Farfel et al., 2016). The relationship between APOE4 carrier status and CSF tau levels is more robustly correlated in women than in men (Hohman et al., 2018), suggesting a possible sex effect in APOE4-mediated toxicity. Neurons expressing P301S tau are less viable when co-cultured with APOE4-expressing glia compared to APOE2- or APOE3-expressing glia, while co-culture with APOE^−/−^ glia leads to the greatest neuronal viability, supporting the idea that APOE4 represents a toxic gain-of-function (Shi et al., 2017). Higher CSF tau levels are associated with faster disease progression and reduced cortical plasticity in patients, but only in APOE4 carriers (Koch et al., 2017), further cementing a role for APOE4 in exacerbating tau pathology. Since some evidence suggests that APOE can be expressed by neurons under stress (Xu et al., 1998, 2006; Harris et al., 2004), it is possible that neuron-derived APOE4 directly mediates tau toxicity in neurons, but the above data suggests that glia-derived APOE4 is likely contributing as well.

### Amyloid- and Tau-Independent Effects of APOE4: Glial Cell Biology

The role of APOE4 in neurological disease is certainly broader than the clearance or response to misfolded proteins, including Aβ and tau; for example, APOE receptors play diverse roles in brain physiology independent of Aβ (Holtzman et al., 2012) and APOE4 carriers may be susceptible to disorders that do not involve proteinopathy, such as chemotherapy-induced cognitive dysfunction (Mandelblatt et al., 2018; Speidell et al., 2019). In addition to the role of APOE4 derived from astrocytes and microglia, a growing body of literature also supports a role for APOE4 and pericytes at the blood brain barrier in neurovascular unit dysfunction and AD pathogenesis (Casey et al., 2015; Soto et al., 2015; Halliday et al., 2016; Ma et al., 2018). APOE has even been proposed to be proteolytically cleaved to form either cytotoxic or neuroprotective fragments, in a cell type- and isoform-specific manner (Brecht et al., 2004; Muñoz et al., 2018). Thus, the neurotoxicity conferred by APOE4 in AD may not be solely due to its effects on amyloid or tau pathology, but also to its effects on normal glial functions. How these processes fit into the current understanding of APOE function and neurodegeneration will be important for drug discovery efforts targeting APOE biology to treat AD.

Astrocytes play critical roles in brain lipid and energy metabolism, and both microglia and astrocytes have important immune functions in the brain. APOE4 expression in each of these cell types likely disrupts these pathways, ultimately leading to brain dysfunction in addition to any Aβ- and tau-mediated effects. The role of APOE4 and aging in each of these cell types and pathways will now be examined individually.

## APOE and Astrocyte Bioenergetics

The idea that APOE isoforms differentially mediate astrocyte bioenergetics has gained increasing support in recent years and implies that APOE4-expressing astrocytes have deficient lipid and glucose metabolism, impairing their ability to support energy-demanding neurons, particularly during aging. In the following sections, we will describe different aspects of lipid homeostasis and glucose metabolism in astrocytes, and how APOE may be involved in these processes.

### Astrocytes and Lipid Homeostasis in the Aging and AD Brain

The most well-studied aspect of APOE biology in AD is lipid transport, which neurons rely upon for their proper function. Lipid homeostasis is clearly altered in AD: in his first description of the disease, Alois Alzheimer noted that “many glial cells show adipose saccules” (Alzheimer et al., 1995), and lipid accumulations are present in both human AD brain and in an AD mouse model (Hamilton et al., 2015), as well as in the aging mouse brain (Shimabukuro et al., 2016). Given that the brain is the most lipid-rich organ outside of adipose tissue (O’Brien and Sampson, 1965), it is therefore not surprising that lipoproteins, cholesterol and lipid homeostasis are critical for normal brain function, including neuronal repair, membrane remodeling, and plasticity (Mahley, 2016). For example, disrupting lipid homeostasis in mice by knocking out both the α and β isoforms of LXR, which are required for cholesterol and lipid efflux from astrocytes, leads to widespread abnormalities in the brain, including an age-dependent accumulation of lipid vacuoles in perivascular astrocytes (Wang et al., 2002). When SREBP2, a major positive regulator of cholesterol and lipid synthesis, is specifically knocked out in astrocytes, mice exhibit reduced brain weight and deficits in social behavior, learning and memory, and coordinated movement, as well as elevated glucose oxidation (Ferris et al., 2017). Interestingly, the neurons in these mice show elevated SREBP2, possibly to compensate for the lack of SREBP2 in astrocytes; yet this neuron-specific SREBP2 elevation was not enough to rescue the pathological changes associated with astrocyte-specific knock-out, underscoring the dependence of neurons on astrocytic lipids.

**Figure 2:**
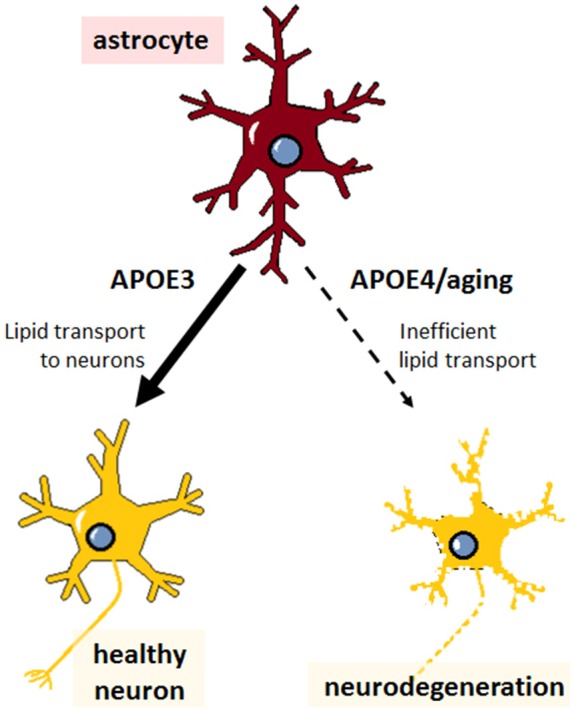
Impaired lipid transport capacity of astrocytic APOE4 sensitizes neurons to degeneration during aging. Astrocytes (red) expressing APOE3 supply normal levels of cholesterol and other lipids to the cells of the brain, particularly to neurons (yellow), maintaining healthy neuronal function and cognition. Astrocytes expressing APOE4 are less inefficient at lipid transport, which, compounded with aging-associated lipid dysregulation, leads to neurodegeneration (neuron with rough edges) and is expected to predispose APOE4 carriers to Alzheimer’s disease (AD).

APOE4 from primary astrocytes is poorly lipidated compared to APOE3 (Gong et al., 2002); deficient lipid binding and transport by APOE4 might therefore be expected to result in the same type of widespread brain abnormalities described above, ultimately leading to increased risk for AD (Figure 2). But despite the poor lipid transport capabilities of APOE4 and the reliance of neurons on astrocyte-supplied lipid, APOE4 carriers have generally normal brain function throughout life. How then does aging uncover the deficits conferred by APOE4? The young brain may have mechanisms in place to cope with inefficient APOE4 lipid transport; but aging leads to decreased cholesterol synthesis in astrocytes (Boisvert et al., 2018), which, when combined with lower efflux from APOE4, could tip the balance and culminate in neuronal lipid deficits. Furthermore, Aβ inhibits SREBP2 in primary cultured cells from mouse cortex (Mohamed et al., 2018), suggesting that amyloid deposition could make neurons even more dependent on astrocytic lipids, which would be lacking in ε4 carriers. Although cholesterol has been the most extensively studied, changes in other lipid classes are also observed in serum samples from AD patients, including sterols, sphingomyelin, phosphatidylcholine, glycerophosphoethanolamine, lysophosphatidylcholine, diacylglycerols, and triacylglycerols (Anand et al., 2017); therefore, APOE4 status could exacerbate other age-related changes in lipid homeostasis. While it is unclear whether these changes in lipid metabolism are a cause or an effect of AD, aging- and APOE-related perturbations may be expected to exacerbate amyloid pathology, and vice versa, culminating in widespread neurodegeneration.

### Paradoxical Effects of APOE4 on Cholesterol Synthesis

Given that APOE4-containing lipoproteins are lipid-deficient, one might expect lipid secretion to be impaired. Surprisingly, human iPSC-derived astrocytes expressing APOE4 reportedly secrete *significantly more* cholesterol than their APOE3+ counterparts (Lin et al., 2018). Notably, this enhanced cholesterol secretion was accompanied by higher, not lower, intracellular cholesterol. Accumulated intracellular cholesterol is consistent with the reduced ability of APOE4 to export cholesterol, which was confirmed in an independent study showing that APOE4 iPSC-derived astrocytes produce APOE-lipoprotein particles with less cholesterol than APOE3-expressing cells (Zhao et al., 2017a). But the increased cholesterol secretion is more difficult to explain; how could APOE4 promote both the intracellular accumulation and enhanced extracellular secretion of cholesterol *in vitro*? One explanation to unite these seemingly contradictory findings is that these cells are unable to properly sense that intracellular cholesterol levels are high. Normally, negative feedback loops ensure that cells laden with lipids reduce synthesis and uptake while increasing efflux—consistent with this signaling mechanism, SREBP2 was in fact downregulated in APOE4+ iPSC-derived astrocytes, as would be expected from cells with excessive lipids (Lin et al., 2018). APOE4 might therefore reduce the clearance of cholesterol *via* enzymatic oxidation. Consistent with this idea, APOE^−/−^ mice have reduced 24-OH-, 7α, and 7β-hydroxycholesterol in their brains (Nunes et al., 2018). Furthermore, *APOE* mRNA levels are reduced in the APOE4+ iPSCs, and *APOE* is a major target gene of LXRs, which are activated by hydroxycholesterol. In contrast to the above finding, astrocytes from targeted-replacement mice expressing APOE4 were previously found to secrete less cholesterol than astrocytes from APOE3 mice (Gong et al., 2002; Riddell et al., 2008). While it is possible that species differences in cholesterol handling between mice and humans could explain these disparate findings (Dietschy and Turley, 2002), more research is needed to clarify exactly how APOE genotype affects astrocyte cholesterol metabolism.

### APOE4 Disrupts Lipid Droplet Homeostasis

**Figure 3:**
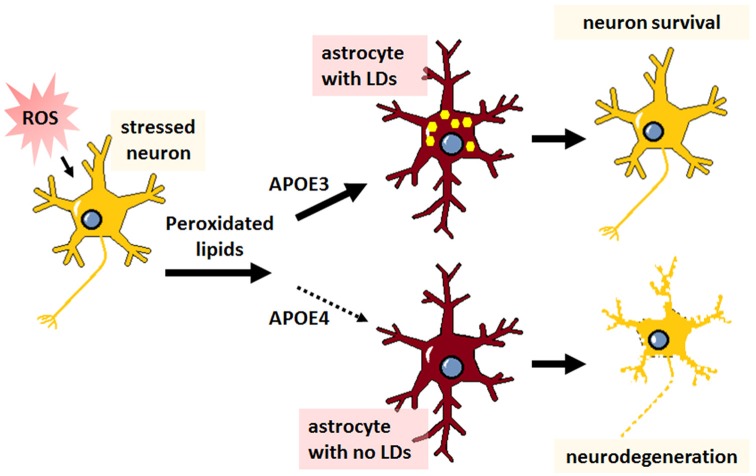
The neuroprotective transfer of toxic lipids from neurons to astrocytes results in lipid droplet formation, which is abrogated in APOE4-expressing cells. Agents or stressors that induce reactive oxygen species (ROS) formation in neurons leads to increased levels of toxic peroxidated lipids. Neurons transfer these lipids to astrocytes *via* APOE. With APOE3 expression, this transfer results in the formation of lipid droplets (LDs; yellow dots) in astrocytes and neuroprotection. Conversely, APOE4 expression is thought to prevent the transfer of peroxidated lipids to astrocytes, resulting in no LD formation in astrocytes and subsequent neurodegeneration.

Recent observations indicate that APOE regulates intracellular lipid storage. A consequence of SREBP2 inhibition, as might occur during aging or amyloid deposition, is the reduction of autophagic lipid mobilization from structures known as lipid droplets (LDs; Seo et al., 2011; Kim et al., 2016). LDs are intracellular accumulations of neutral lipids and are central to cellular lipid homeostasis, particularly in astrocytes, where they play a dual role in managing lipids from neurons and in maintaining astrocytic energy demands. Elevated reactive oxygen species (ROS) in neurons induces lipid peroxidation and triggers subsequent efflux of lipids that accumulate as LDs in neighboring astrocytes, a process that is neuroprotective and dependent on APOE (Figure 3; Liu et al., 2015, 2017b). An increase in peroxidated lipids is associated with disrupted lipid homeostasis, decreased phosphatidylcholine synthesis, decreased mitochondrial metabolism, and ultimately cognitive decline (McDougall et al., 2017), and APOE mitigates this toxicity in neurons by transferring the burden of lipid accumulation and subsequent clearance to astrocytes. In a *Drosophila* model of neurodegeneration, APOE4 is a complete loss of function in terms of the neuroprotective formation of LDs in glial cells, leading to neuronal cell death (Figure 3; Liu et al., 2015, 2017b). These data indicate that APOE might play an important role not only in astrocyte-mediated synthesis and transfer of lipids to neurons, but in reverse as well, as an acceptor of neuronal-derived peroxidated lipids.

Although these studies show that APOE4 expression leads to a decrease in LD formation when neurons are the lipid donor and astrocytes are the recipient, APOE4 can also induce LDs in a cell autonomous manner. LD formation results from interactions between the endoplasmic reticulum (ER) and mitochondria, at structures known as mitochondria-associated ER membranes, and APOE may regulate LD formation by mediating ER-mitochondria communication at these sites (Tambini et al., 2016). Fibroblasts treated with APOE4 astrocyte conditioned medium (ACM) exhibit increased LDs, and blocking ER-mitochondria tethering returns lipid levels to normal (Tambini et al., 2016). These data indicate that either the APOE4 ACM contained a factor that signaled to the cells to induce LDs, or alternatively, that the APOE4 ACM is somehow nutrient-deprived compared to APOE3 media, since glucose deprivation also induces LD formation in astrocytes. Nutrient deprivation-induced LDs are used for β-oxidation of fatty acids to generate acetyl-CoA to meet cellular energy demands (Cabodevilla et al., 2013). Thus, it is possible that APOE regulation of LDs in astrocytes is context-dependent: nutrient deprivation induces formation of LDs and subsequent breakdown by autophagy to fulfill energy requirements, which APOE4 can stimulate; whereas increased neuronal oxidative stress leads to accumulation of toxic lipids, which are transferred to astrocytes, an activity that is lacking in APOE4 cells. In either case, APOE4-dependent deficiency in autophagy would also impair LD breakdown, causing toxic accumulation in either cell type (Simonovitch et al., 2016). Further study delineating the impact APOE4 has on LD homeostasis could identify points of therapeutic intervention.

### Astrocyte Glucose Metabolism in the Aging Brain

The brain is a highly energy-demanding organ, and declines in brain glucose utilization and mitochondrial function during aging may interact with AD risk factors, including APOE, to negatively impact neuronal homeostasis. The data supporting this concept range from model organisms to epidemiology. For example, yeast genes that enhance or suppress Aβ toxicity exert their effect depending on the level of mitochondrial respiration (Treusch et al., 2011), suggesting that cellular energetics determines resiliency to amyloid. Energetics also impacts AD risk profile in humans: postmenopausal women characterized as having a poor metabolic profile, which includes elevated glucose and increased insulin resistance, exhibit worse cognitive performance compared to healthy metabolic subjects, and cognitive decline in this group is exacerbated by APOE4 carrier status (Karim et al., 2019). However, this relationship is likely complex; a study including both aged women and men found no difference in glucose levels in AD and APOE4 carriers vs. healthy and non-APOE4 carriers; there were marginal reductions in insulin and insulin resistance in APOE4 carriers, which was somewhat increased in individuals with AD (Morris et al., 2017).

To clarify the underlying relationship between APOE4 and energy homeostasis, APOE4 targeted-replacement mice have been studied. Aged (22 months) mice expressing APOE4 exhibit decreased insulin signaling in cortex and hippocampus (Zhao et al., 2017b) and middle-aged (6 months) female APOE4 mice are deficient in the uptake and utilization of glucose in the brain, with compromised respiratory capacity and decreased PPARγ signaling (Wu et al., 2018). In addition to downregulated PPARγ, another study found that insulin-degrading enzyme (IDE) was also downregulated in the hippocampus of the same aged APOE4 mice (Keeney et al., 2015). Reduction of PPARγ would be expected to trigger lipid dysregulation by decreasing lipid synthesis (as described above), as well as dampen anti-inflammatory signaling; and while lower levels of IDE would be expected to decrease Aβ clearance, lower IDE should also increase insulin and affect glucose and glycogen levels, perhaps leading over time to insulin resistance, although this would need to be determined experimentally. In the same study, aged mice expressing either APOE4 or APOE3 compared to the neuroprotective APOE2 were also found to have downregulated insulin signaling proteins IGF1, IRS1, and GLUT4 (Keeney et al., 2015), in agreement with the idea that aging itself causes deficient glucose metabolism independently of APOE genotype. As discussed above, energy deficiencies might be exacerbated in APOE4 carriers as lipid β-oxidation and lipid droplet autophagy become increasingly important for cell function.

### Aerobic Glycolysis

**Figure 4:**
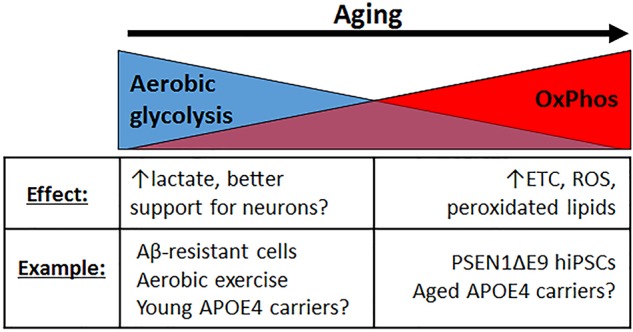
Different modes of glucose metabolism are preferred in the young vs. aging brain. In the young brain, aerobic glycolysis is generally favored, resulting in increased lactate production, presumably by astrocytes, which should be supportive for increased neuronal activity. With aging, there is a shift toward oxidative phosphorylation (OxPhos) instead, resulting in increased electron transport chain (ETC) activity, increased ROS production, and more peroxidated lipids. Healthy cultured cells that are resistant to Aβ toxicity happen to exhibit a preference for aerobic glycolysis, and aerobic exercise, which is known to confer neuroprotection, elevates aerobic glycolysis. In contrast, cells from familial AD patients (PSEN1ΔE9) exhibit elevated OxPhos. While young APOE4 carriers may exhibit increased glycolytic activity, particularly in brain regions associated with AD [e.g., default mode network (DMN), entorhinal cortex], aged APOE4 carriers may exhibit elevated OxPhos instead, although more evidence to demonstrate whether and how such a metabolic shift occurs is warranted.

Deficits in energy metabolism associated with APOE4 might also exacerbate aging-associated declines in aerobic glycolysis (Goyal et al., 2017; Figure 4). Aerobic glycolysis is the preferential conversion of glucose to lactate rather than pyruvate, even in the presence of oxygen, and is typically associated with cancer cells, although non-cancerous cells also engage in this process (Jones and Bianchi, 2015). In fact, astrocytes in mice are capable of surviving solely by aerobic glycolysis for at least as long as 1 year without any signs of pathology or neurodegeneration (Supplie et al., 2017). Certain brain regions tend to preferentially use aerobic glycolysis (Vaishnavi et al., 2010), and as the brain ages, there is a shift towards oxidative phosphorylation (OxPhos) to meet energy requirements (Figure 4; Goyal et al., 2017). This aging-related increased reliance on OxPhos has been proposed to lead to elevated ROS and peroxidated lipids (Harris et al., 2014), a situation likely made worse in APOE4 carriers, given the reduced ability of APOE4 to traffic neuronal peroxidated lipids to astrocytes for elimination. Thus, the switch to OxPhos could be an age-dependent trigger for APOE4 pathophysiology.

In line with the concept that aerobic glycolysis is beneficial or protective, and OxPhos is not, hiPSC-derived astrocytes from AD patients harboring the PSEN1 ΔE9 mutation are more oxidative than isogenic controls, with increased ROS production and decreased lactate secretion (Oksanen et al., 2017). On the other hand, PC12 and B12 cells that are resistant to Aβ toxicity exhibit upregulated aerobic glycolysis (Newington et al., 2011). Furthermore, aerobic exercise, which improves cognitive scores in aging and AD patients (Panza et al., 2018), increases aerobic glycolysis and lactate production in the brain (Matsui et al., 2017). Interestingly, in the entorhinal cortex of APOE4 targeted-replacement mice, genes involved in OxPhos are upregulated, suggesting an APOE4-dependent increase in OxPhos and decrease in aerobic glycolysis and lactate (Figure 4; Nuriel et al., 2017b). Therapeutic strategies aimed at improving aerobic glycolysis may therefore help ameliorate APOE4-mediated toxicity.

### Glycogen in Astrocytes

Despite its high energy demands, there are few energy stores in the brain compared to the rest of the body. In addition to storing lipids in the form of LDs, astrocytes are also the primary cell type in the brain to store glycogen. Astrocytic glycogen is important for maintaining healthy neurons and overall brain function, providing an energetic buffer during periods of low glucose availability (Bak et al., 2018). Primary astrocytes cultured in high (25 mM) vs. low glucose (5.5 mM) have elevated rates of glycolysis and glycogen content (Li et al., 2018). Elevated glycogen stores in co-cultured astrocytes are neuroprotective during glucose deprivation (Swanson and Choi, 1993). Significant evidence connects glycogen with memory formation: mice lacking glycogen synthase in the brain have impairments in learning- and memory-associated synaptic plasticity (Duran et al., 2013); glycogenolysis is important for memory consolidation (Gibbs et al., 2006); glycogen is a precursor to glutamate for learning (Gibbs et al., 2007); and glycogen content changes with early memory consolidation in 1-day-old chick (Hertz et al., 2003). Activated glycogen synthase kinase 3 has long been associated with the hallmarks of AD, including Aβ deposition, tau hyperphosphorylation, and brain inflammation, and would furthermore be expected to inhibit glycogen synthesis and thus decrease glycogen stores (Rayasam et al., 2009).

Energy metabolism in the brain may change during aging in a cell type-dependent manner. Inhibition of glycogen breakdown, termed “glycogenolysis,” disrupts long-term potentiation in young, but not old, rat hippocampus (Drulis-Fajdasz et al., 2015). In a follow-up proteomics study, the same group found that, while glycogen phosphorylase (PYGB), the rate-limiting enzyme in glycogen degradation, is predominantly expressed in astrocytes in young animals, its distribution switches to being present in both neurons and astrocytes in old animals (Drulis-Fajdasz et al., 2018). As glycogen accumulation in neurons normally triggers apoptosis (Vilchez et al., 2007; Duran et al., 2012), the authors speculate that upregulation of PYGB in neurons may be a protective mechanism to keep neuronal glycogen stores low. However, total depletion of glycogen in neurons may not be desirable in all circumstances, as low levels of neuronal glycogen may be protective during hypoxia (Saez et al., 2014).

The importance of glycogen to enhanced memory is not necessarily ascribed to elevated pyruvate for mitochondrial OxPhos, since aged wild-type and adult APP/PS1 mice fed a diet supplemented with pyruvate still exhibit impairments in a passive avoidance task for fear memory, despite a preservation of glycogen stores and enhanced exploratory behavior (Koivisto et al., 2016). Rather than supplying pyruvate for OxPhos, glycogen may instead supply lactate to mediate its beneficial effects, as described in the following section.

### Lactate, Glycogen and the Astrocyte-Neuron Lactate Shuttle Hypothesis

The Astrocyte-Neuron Lactate Shuttle (ANLS) hypothesis was first formulated in 1994 (Pellerin and Magistretti, 1994, 2012), and describes a process in which astrocytes metabolize glucose to export lactate for neurons during periods of high neuronal activity, during learning and memory, for example. The existence of an astrocyte-to-neuron transport of lactate would necessitate a lower basal concentration of lactate in neurons compared to astrocytes, and a recent study has indeed demonstrated such a gradient *in vivo*, using a genetically-encoded lactate sensor (Machler et al., 2016).

Despite findings in support of the ANLS hypothesis, there has been some disagreement in the field as to whether astrocytic lactate is really used by active neurons in the brain, if neurons are able to produce their own alternative energy substrates, or if astrocytes produce lactate in response to their own energetic demands (Dienel, 2012). For example, computer simulations of neuron/astrocyte energetics, based on fMRS data, support a model in which neurons readily metabolize glucose and export lactate, which is taken up by astrocytes, and not the other way around (Simpson et al., 2007; Mangia et al., 2009).

Although neurons are certainly capable of taking up glucose and secreting lactate themselves, there is compelling evidence that lactate secretion from astrocytes, derived from glycogen stores specifically, is important in contexts of neuronal high energy demand. For example, the transport of lactate specifically from astrocytes to neurons is necessary for long-term memory formation (Suzuki et al., 2011) and spatial working memory (Newman et al., 2011). Neuronal activity can upregulate astrocytic genes involved in lactate production and export (Hasel et al., 2017), ensuring that astrocytes are able to supply neurons with the necessary lactate during periods of intense energetic demands. Lactate derived from astrocytic glycogen can sustain neuronal activity in the absence of other forms of energy, and blocking the transfer of lactate from astrocytes to neurons in the absence of any other energy source leads to axonal/neuronal failure (Ransom and Fern, 1997; Wender et al., 2000; Brown et al., 2003, 2005; Suh et al., 2007; Walls et al., 2008). Blocking glycogen degradation or lactate transfer reduces glutamate release from neurons (Sickmann et al., 2009). Furthermore, exhaustive exercise decreases brain glycogen and elevates astrocyte-derived lactate (Matsui et al., 2017). While the original ANLS hypothesis may undergo revision and refinement, astrocytic glycogen-derived lactate certainly appears to be an important component of healthy neuronal function, particularly during times of nutrient deficiency.

### Connecting APOE4 and Brain Energy Metabolism: Future Directions

How could aging glycogen metabolism interface with APOE4 genotype to exacerbate neurodegeneration? Young adult APOE4 carriers have altered expression of proteins involved in glucose metabolism in the posterior cingulate cortex (PCC), a central component of the DMN (Perkins et al., 2016). Subregions of the PCC are proposed to be involved in internally directed cognition, including memory retrieval and planning, as well as controlling attentional focus (Leech and Sharp, 2014). The DMN is highly metabolically active and is one of the earliest regions to deteriorate in AD and in normal aging (Leech and Sharp, 2014), and young APOE4 carriers exhibit increased activity in the DMN before any signs of disease (Filippini et al., 2009). In agreement with this increased activity, hiPSC-derived neurons from APOE4 patients are hyperactive (Lin et al., 2018), and APOE4 targeted-replacement mice exhibit a hyperactive entorhinal cortex compared to APOE3-expressing mice (Nuriel et al., 2017a). While young APOE4 carriers were found to express higher levels of glycolysis enzymes (GLUT1, GLUT3, HEX1, MCT2, SCOT, AACS) and complexes I, II, and IV of the electron transport chain (ETC), there were lower levels of MCT4, an important transporter for astrocytic lactate secretion (Perkins et al., 2016). Disruption of MCT4 impairs long-term memory, which is rescued by lactate injection, while memory impairment caused by disruption of the neuronal lactate transporter MCT2, is not rescued by lactate, strongly supporting the notion that astrocytic export of lactate is critical for long-term memory formation (Suzuki et al., 2011). Thus, the decreased MCT4 in young APOE4 carriers might be expected to cause a deficit in lactate secretion by astrocytes, despite higher glycolytic activity. Interestingly, the DMN is a region that relies on aerobic glycolysis in young, healthy brain (Vaishnavi et al., 2010), and so should be a region that relies on elevated lactate production to support neuronal activity; the increased neuronal activity and decreased capacity of astrocytes to keep up with such activity in APOE4 carriers might then be expected to burn out glycogen stores early, effectively accelerating an aging-associated metabolic phenotype reliant on OxPhos. Further work is necessary to determine whether this pathway could be induced by diet, exercise, or pharmacological intervention to preserve cognitive function in presymptomatic APOE4 carriers.

In summary, APOE performs a complex set of interrelated functions in astrocytes, ranging from its long-appreciated lipid transport function to regulation of lipid storage and utilization to cellular energetics. In the next section we will review emerging concepts around APOE function in the other major producer of APOE in the brain, microglia.

## Microglia-Derived APOE in Aging and AD

A large body of evidence implicates microglia in APOE-mediated AD pathogenesis, particularly in relation to aging. Microglial APOE production is strongly induced during injury and disease, including in AD (Olah et al., 2018; Ping et al., 2018; Rangaraju et al., 2018a). In 5XFAD transgenic mice, which harbor five different human familial AD-causing mutations and exhibit accelerated amyloid pathology (Oakley et al., 2006), microglial APOE mRNA is significantly increased (Wang et al., 2015). A similar increase in microglial APOE mRNA was also found in a separate but similar transgenic mouse model of accelerated amyloid pathology, APP/PS1 (Orre et al., 2014), as well as in aged (isolated from 24 month old mice) vs. younger (5 month old) mouse microglia (Hickman et al., 2013), and in the Ercc1 mutant mouse model of accelerated aging (Raj et al., 2014a; Holtman et al., 2015). The upregulation of APOE mRNA in these mouse models of AD and aging reflect concordant increases at the protein level and in human AD brain. A recent proteomics study of microglia isolated from 5XFAD mice identified APOE as one of the top upregulated proteins (Rangaraju et al., 2018a). Interestingly, immunohistochemical analysis in this same study indicated that the microglia with elevated APOE were those surrounding amyloid plaques, demonstrating that a distinct subset of microglia increase APOE expression, rather than all microglia. Furthermore, the aged mouse microglial proteome also shows an enrichment in APOE protein compared to non-aged mice (Rangaraju et al., 2018a). In agreement with these findings in mice, an analysis of frontal cortex human postmortem brain tissue found elevated APOE protein in AD patients vs. healthy controls (Ping et al., 2018). Another study performing a post-mortem human brain proteomics analysis also found APOE to be higher in the aged microglia (Olah et al., 2018). Thus aging alone, and not only disease pathogenesis, is sufficient to induce microglial APOE expression at both the mRNA and protein level.

**Figure 5:**
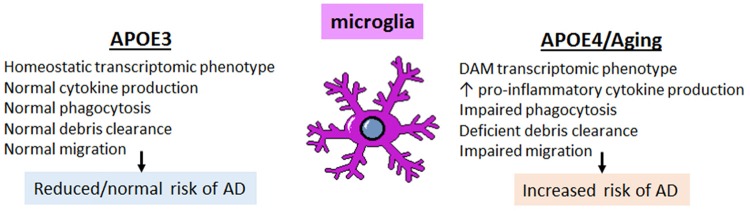
The effect of APOE4 expression on microglia is cell autonomous and triggers a DAM, pro-inflammatory phenotype with impaired homeostatic functions. Microglia expressing APOE4 vs. APOE3 tend to exhibit a disease-associated microglia (DAM)-like phenotype; this includes increased pro-inflammatory cytokine production with impaired phagocytic ability, deficient clearance of debris (including amyloid plaques), and impaired migratory ability. These changes, compounded with the pro-inflammatory phenotype associated with normal aging, result in an increased risk of developing AD.

The expression of APOE in subsets of disease- and aging-associated microglia raises an important question: what role does APOE play in the microglial response to disease and aging, and how is this impacted by APOE4 genotype? Both mouse and human studies indicate that key microglial functions are affected by APOE genotype, including transcriptomic changes towards the disease-associated phenotype, the percent of microglia coverage around plaques, increased cytokine production, as well as chemotaxis, phagocytosis and, perhaps, synaptic pruning (Figure 5). Each of these functions are discussed in the following sections.

### APOE and the Microglial Phenotype in AD

The transcriptional profile of microglia is altered in AD, switching from a homeostatic phenotype to a molecular profile often referred to as the disease-associated microglial (DAM) phenotype (Zhang et al., 2013; Keren-Shaul et al., 2017; Sarlus and Heneka, 2017; Rangaraju et al., 2018b). Genome-wide association studies (GWAS), including large-scale meta-analyses, have indicated that the majority of genetic variants conferring risk for late onset sporadic AD are immune-related and enriched in microglia, implicating DAM microglia in AD pathogenesis (Guerreiro et al., 2013; Lambert et al., 2013; Dos Santos et al., 2017; Huang et al., 2017). The affected genes include myeloid receptors *TREM2* and *CD33*, transcriptional regulators *SPI1* (Pu.1) and *MEF2C*, complement pathway (*CR1*), antigen presentation (*HLA-DRB5*), the *MS4A* family locus, and *ABCA7*, amongst several others (Lambert et al., 2013). Some single nucleotide polymorphisms (SNPs) are present in non-coding regions and alter expression of microglial genes (e.g., *SPI1*; *CD33*), whereas other SNPs result in a gain or loss of function in several microglial genes related to immune function (e.g., *TREM2*) (Raj et al., 2014b; Malik et al., 2015; Huang et al., 2017). A large-scale weighted gene coexpression network analysis (WGCNA) combined with pathological assessment of 1647 post-mortem brain tissues from late-onset AD patients and non-demented controls pointed to immune/microglial gene networks as having the most significant functional enrichment of all modules (Rangaraju et al., 2018b). Moreover, this microglial module was significantly associated with the greatest number of AD-relevant pathological traits, including the extent of brain atrophy, and represented immune pathways consisting of complement, Fc-receptors, major histocompatibility complex (MHC), cytokines/chemokines and toll-like receptors (Zhang et al., 2013). Since then, several groups have characterized this DAM phenotype/immune network, albeit with varying nomenclature (Gjoneska et al., 2015; Keren-Shaul et al., 2017; Rangaraju et al., 2018b), and attempts to identify key regulators of this transcriptomic phenotype have been underway (Gjoneska et al., 2015). A common theme on which these genetic risk factors converge is that they alter key microglia activities, including phagocytosis, cytokine production, and microglial encapsulation of amyloid plaques (Figure 5). Altogether, this work has repositioned the thinking in the field, emphasizing microglia as a potential source of attractive therapeutic targets for AD.

Interestingly, APOE is a key regulator of the microglial transcriptional signature, as demonstrated in post-mortem human brain studies, human cellular models as well as in AD mouse models and cultured microglia *in vitro* (Keren-Shaul et al., 2017; Krasemann et al., 2017; Pimenova et al., 2017; Lin et al., 2018; Olah et al., 2018). Studies performing single cell RNA sequencing of CD45+ microglia from 5XFAD mice, paired with *in situ* hybridization of DAM signature genes, indicate that both the morphology and molecular identity of microglia around plaques, as a population, are different from microglia distal to plaques (Keren-Shaul et al., 2017). These DAMs have increased APOE expression that is triggered following a downregulation in homeostatic genes such as CX3CR1 and P2Y12 (Keren-Shaul et al., 2017; Krasemann et al., 2017). Furthermore, APOE mediates the switch from homeostatic to the DAM phenotype; notably, knocking out APOE specifically in microglia in 5XFAD mice prevents the transition to the DAM phenotype and partially rescues neuronal cell death in an axotomized facial motor nucleus model (Krasemann et al., 2017). A full knockout of APOE conferred no additional protection over that of microglia-selective APOE deletion, highlighting the importance of microglia-specific APOE to this process (as opposed to astrocytic APOE, for example; Krasemann et al., 2017). However, an astrocyte-selective APOE model was not directly compared, so it remains possible that it is not the cellular source of APOE that matters, but rather a reduction in total APOE levels that underlies this finding.

While elucidating the role of mouse APOE is informative, it is also critical to understand whether different phenotypes ensue with human APOE variants. Microglia isolated from aged vs. non-aged human postmortem brain for RNA sequencing analysis display an immune-enriched signature that is significantly associated with key traits, including APOE genotype (Olah et al., 2018). Although statistical significance was not reached for APOE4, the neuroprotective APOE2 was associated with a reduction in this aged microglial phenotype (Olah et al., 2018). In human cellular models, isogenic conversion of human iPSC-derived microglia from APOE3/E3 AD patients to APOE4/E4 is sufficient to transform the microglia transcriptome to a DAM-like phenotype (Lin et al., 2018). Notably, this APOE4 gene expression signature significantly overlapped with the transcriptional profile seen in human brain (Lin et al., 2018), in support of the notion that APOE4 may impact the DAM phenotype in human AD. A WGCNA transcriptomic analysis of brain from APOE3 or APOE4 targeted-replacement mice subjected to traumatic brain injury identified that the network most significantly associated with APOE genotype was the “innate immune response,” which included complement activation; in this network, the genes were shifted toward increased expression along with APOE4 compared to APOE3 (Castranio et al., 2017), again supporting a model in which APOE4 confers a pro-inflammatory phenotype relative to APOE3.

### APOE Regulation of Microglial Plaque Association

APOE immunoreactivity in human brain is enriched in congophilic, dense-core plaques (Navarro et al., 2003), as opposed to diffuse plaques, which are heterogeneous with respect to APOE immunoreactivity (Gearing et al., 1995). Notably, microglial activation around diffuse plaques is minimal (Maat-Schieman et al., 1994; Stalder et al., 1999; Mrak, 2012), begging the question as to whether the presence of APOE in plaques is the trigger that differentially activates microglia at specific plaque types, or whether the presence of APOE in the plaques is simply due to the upregulation of APOE upon transition from homeostatic microglia to DAMs (Ulrich et al., 2014; Krasemann et al., 2017).

APOE4-expressing immune cells are less efficient at plaque engulfment compared to APOE3-expressing cells. When GFP+ bone marrow cells from human *APOE3* or *APOE4* donor mice were transplanted into lethally-irradiated 5 month old *APPswe/PS1ΔE9* [i.e., bone marrow transplanted (BMT)-APP/PS1] mice, donor GFP+ macrophages are found in the brain 8 months later, with APOE3 exhibiting greater numbers of plaque-associated GFP+ Iba1+ cells (Yang et al., 2013). Interestingly, APOE4 was associated with reduced microglia coverage around Aβ plaques (Yang et al., 2013). Proper microglial encapsulation of plaques is thought to be protective, sequestering damage from surrounding cells, and decreased microglial coverage is associated with higher Aβ levels and increased neuronal dystrophy (Yeh et al., 2016). Indeed, the percentage of Aβ per area was significantly higher in the hippocampus and cortex of mice with APOE4 vs. APOE3 transplant (Yang et al., 2013). Furthermore, APOE4 BMT-APP/PS1 mice had significantly higher brain expression levels of the pro-inflammatory genes TNFα and macrophage migration inhibitory factor (MIF; which are upregulated in AD patients), lower levels of the anti-inflammatory gene IL-10, and impaired spatial working memory in the Barnes maze, compared with *APOE3* BMT-*APP/PS1* mice (Yang et al., 2013).

In another study using 5XFAD mice crossed to APOE3 or APOE4 targeted-replacement mice, mice expressing APOE4 exhibited significantly larger and more numerous amyloid plaques, as well as increased microglial dystrophy; but in contrast to the Yang et al. (2013) study, more microglia were found surrounding plaques in APOE4 vs. APOE3 and APOE2 (Rodriguez et al., 2014). It is difficult to distinguish whether the change in microglia phenotype in relation to plaque type is indirect, in response to worsened pathology or if it is also partly due to a cell autonomous effect of APOE4 on microglia, irrespective of plaque type. While these *in vivo* studies are informative, other recent studies indicate cell-intrinsic APOE4 effects on microglia. More specifically, human iPSC-derived microglia from APOE4 carriers have different morphology compared to isogenic APOE3 controls, and have a reduced capacity to phagocytose Aβ (Lin et al., 2018), in agreement with a change towards the DAM phenotype. Thus, APOE4 expression impairs the ability of microglia to efficiently clear amyloid pathology, although the precise mechanisms underlying microglial recruitment to specific amyloid plaques require further characterization.

### APOE Genotype and Cytokine Production

An overwhelming body of evidence supports that the presence of APOE4, either recombinantly applied or endogenously expressed, confers an increase in pro-inflammatory cytokine production across rodent and human species, in blood, brain, and microglia. In support, rat primary glial cultures comprised of astrocytes and microglia produce higher levels of IL-1β when exposed to recombinant APOE4, purified from APOE-expressing HEK293 cell culture medium, than APOE3 (Guo et al., 2004). Cultured mouse microglia derived from APOE4 targeted-replacement mice have an activated morphology, produce higher levels of pro-inflammatory cytokines including TNFα, IL-6, and IL12p40, and nitric oxide (NO) along with lower levels of anti-inflammatory cytokines than their APOE3-derived counterpart when exposed to various pro-inflammatory mediators including LPS, IFNγ, or LPS+ IFNγ (Brown et al., 2002; Colton et al., 2005; Vitek et al., 2009). Notably, some of these effects (e.g., NO production) are APOE4 gene dosage-dependent (Vitek et al., 2009).

Similar to that seen in cultured microglia, APOE4 mice immune-challenged with a peripheral injection of LPS exhibit higher brain mRNA expression levels of TNFα and IL12p40 than in that from APOE3 TR mice (Vitek et al., 2009). A similar increase in pro-inflammatory cytokines, namely TNFα and IL-6, in APOE4 mouse serum is seen following a peripheral injection with LPS compared to that in APOE3 mice (Lynch et al., 2003). Finally, when LPS is administered by intracerebroventricular injection, APOE4 mice have higher brain levels of IL-1β, IL-6, and TNFα than APOE3 mice (Zhu et al., 2012).

While this increase has been consistently observed by independent groups *in vivo* in APOE targeted-replacement mice in AD models (Tai et al., 2011), due to perhaps independent roles of APOE genotype on other aspects of the disease (e.g., Aβ plaque levels), it is not clear whether the increase in cytokines by APOE4 is due to the increase in pathology, or due to a direct effect of APOE4 on cytokine production, which could contribute to the increase in pathological changes. Cell culture experiments shed some light on the former, in that the effect of APOE4 seems to be a cell-autonomous effect on microglia as when stimulated in culture, they produce more pro-inflammatory cytokines such as IL-1β (Guo et al., 2004), which suggests the differential extent of pathology (e.g., amyloid plaque deposition) as not being the sole driver of the differential increase in cytokines due to APOE4 vs. APOE3 genotype. Since these APOE targeted-replacement mouse studies assess the effect of human APOE in a mouse context, it remains plausible that this toxic pro-inflammatory effect attributed to APOE4 could be specific to mouse; however, human data indicates otherwise and suggests this phenomenon is intrinsic to the human APOE isoform irrespective of species by which it is produced/acting upon. Indeed, over the past few years, studies using advanced human cellular models parallel the pro-inflammatory findings seen in mice (Lin et al., 2018). Further, human clinical data suggests something similar. In two Chinese populations with AD, APOE4 carriers, carrying either one or two copies, had elevated plasma levels of the pro-inflammatory cytokines TNF-α, IL-6, and IL-1β compared to that of APOE2 and APOE3 carriers (Fan et al., 2017). Also, APOE genotype modulates cytokine production in human peripheral blood when stimulated with pro-inflammatory mediators *ex vivo as* well as *in vivo*. More specifically, *ex vivo* stimulation of peripheral blood collected from healthy volunteers with TLR2 and TLR4 ligands demonstrated that TNFα, IL-1β, IL-6, IL-17, IFNγ, G-CSF, IL-8, MCP-1, MIP-1a, and IP-10 levels were robustly increased in that from APOE3/E4 compared to APOE3/E3 carriers (Gale et al., 2014). Similarly, healthy human subjects intravenously administered the TLR4 ligand LPS exhibited higher plasma TNFα levels in APOE3/E4 vs. E3/E3 (Gale et al., 2014). In recent years, more advanced human cellular models make the picture clearer and indicate the mouse findings are not species specific and extend to human microglia.

Are these effects good or bad? Notably, recent studies have been controversial as to whether the best therapeutic approach for AD with respect to targeting APOE would be to lower APOE levels or augment them. As in astrocytes, the effects of APOE4 in microglia are often confounded by reports that APOE4 production and/or protein stability is lower compared to APOE3 (Bertrand et al., 1995; Raffai et al., 2001; Glockner et al., 2002). Thus, it remains plausible that this inflammatory response could be due to a decrease in APOE levels, irrespective of genotype. APOE3 can dampen cytokine production, and removing APOE can lead to a more pro-inflammatory phenotype. So, would elevating APOE4 protein levels help ameliorate the pro-inflammatory phenotype, or worsen it? Microglial APOE is neuroprotective in rat microglia neuronal co-cultures (Polazzi et al., 2015) and this release of APOE and the resulting neuroprotective effect is lost when microglia are exposed to inflammatory stimuli, thus lowering APOE. Therefore, it is interesting to hypothesize that, in the context of AD, when microglia are exposed to pro-inflammatory stimuli, APOE synthesis and secretion is stunted (Saura et al., 2003; Polazzi et al., 2015), effectively decreasing any neuroprotective effects of the microglia. A study examining the effect of APOE genotype comparing WT neurons cultured with either APOE3 vs. APOE4 mouse-derived astrocytes or microglia found that only APOE4 microglia led to greater neurotoxicity (Maezawa et al., 2006). Interestingly, the greater toxicity of APOE4 correlated with higher pro-inflammatory cytokine levels (TNFα, IL-6, IL-1β). Finally, it should be noted that the APOE4 effects can be sex-specific in certain contexts (Colton et al., 2005).

### APOE Effect on Phagocytosis, Synaptic Pruning, and Chemotaxis

While the effect of APOE genotype on synaptic pruning and phagocytosis has been not been studied in microglia, astrocytic phagocytosis has been evaluated using APOE2, APOE3, and APOE4 targeted-replacement mice crossed to mice expressing EGFP driven by the astrocyte-specific promoter *Aldh1l1* (Chung et al., 2016). Fluorophore-conjugated cholera toxin-β subunit (CTB-594) was used to label axonal projections of retinal ganglion cells and the dorsal lateral geniculate nucleus, an area with a high degree of synaptic pruning during development. In agreement with *in vitro* phagocytic assessments, astrocytes in APOE2 mice showed significantly enhanced phagocytic capacity compared with *APOE3*, whereas astrocytes in APOE4 mice demonstrated a significant decrease (Chung et al., 2016). Although this study focused on astrocytes, microglia are key players in synaptic pruning (Paolicelli et al., 2011; Schafer et al., 2012); thus, it would be informative to determine whether the effect of APOE genotype on synaptic pruning is cell type-specific or not. *In vitro*, ApoE^−/−^ mouse-derived peritoneal macrophages demonstrated a decreased uptake of apoptotic cells, but no change in ability to uptake latex beads, compared to WT (Grainger et al., 2004). Given that APOE4 from human CSF was found to form smaller complexes than APOE2 and APOE3, it has been proposed that APOE4 may be deficient in lipid debris clearance, in accordance with phagocytic studies conducted on APOE4 human iPSC-derived microglia (Lin et al., 2018). Finally, microglial migration has also been found to be linked to APOE genotype (Cudaback et al., 2011). Mouse ApoE^−/−^ microglia show reduced ATP- and C5a-triggered migration; likewise, in targeted-replacement mice, APOE2 and APOE4 have reduced ATP- and C5a-triggered migration compared to APOE3 (Cudaback et al., 2011).

### APOE and Other Microglial AD Risk Factors

Several studies have looked at potential interactions between APOE and TREM2, another genetic risk factor for AD (Guerreiro et al., 2013). APOE can bind TREM2 (Atagi et al., 2015; Yeh et al., 2016), and, either directly or indirectly, APOE can alter TREM2 signaling or function (Jendresen et al., 2017). Both APOE and TREM2 are implicated in key steps in the homeostatic to DAM phenotype (Krasemann et al., 2017). It is still unclear whether there is and to what extent there is an interaction in APOE and TREM2, genetically or functionally, warranting further investigation.

Other risk factors for AD have clear functional overlap with APOE. Of note, ABCA7, also expressed in microglia, has been associated with age of onset of AD in a similar manner as APOE. More specifically, the minor allele at rs3764650 in ABCA7 is associated with a delayed onset and shorter disease duration (Kim et al., 2006). While its function in regulating the homeostasis of phospholipids and cholesterol has been the most well studied function of ABCA7 in relation to APOE, it also plays a role in phagocytosis (Tomioka et al., 2017), which has been observed *in vitro* and *in vivo* and been reviewed more extensively elsewhere (Abe-Dohmae and Yokoyama, 2012; Aikawa et al., 2018).

### Future Directions for Understanding Microglial APOE in Immunosenescence

Is microglial APOE upregulation in aging and AD helpful or harmful? Is it a compensatory mechanism, or does it contribute to accelerated aging and neurodegenerative disease pathogenesis? It is interesting to speculate that the two greatest risk factors for late-onset AD, aging and APOE, interact with respect to inflammation, with APOE4 promoting an enhanced inflammatory tone over the course of a lifetime (Olarte et al., 2006; Sando et al., 2008). Microglia undergo senescence with aging, a process termed immunosenescence (Costantini et al., 2018), consistent with a DAM phenotype, and accumulating evidence suggests that APOE4 genotype may aggravate this process to promote neuroinflammation and neurodegeneration in AD. The change in cellular source of APOE from predominantly astrocyte-derived, to astrocyte- and microglia-derived during disease or aging, raises questions as to whether the cellular source of APOE subserves differential functions. It should be noted however, that although APOE immunoreactivity has been demonstrated around plaques in post-mortem human AD brain, co-labeling of APOE with microglial markers has not been investigated. Thus, a careful evaluation of this is warranted given the recent advances in understanding APOE expression in AD models. Finally, although there are clearly centrally mediated and cell-autonomous effects of APOE4, several peripheral effects of APOE4 on immune cells have been observed, and as such, it is unclear as to what extent the peripheral component of APOE4 status has on the risk to AD.

## Conclusion

**Figure 6:**
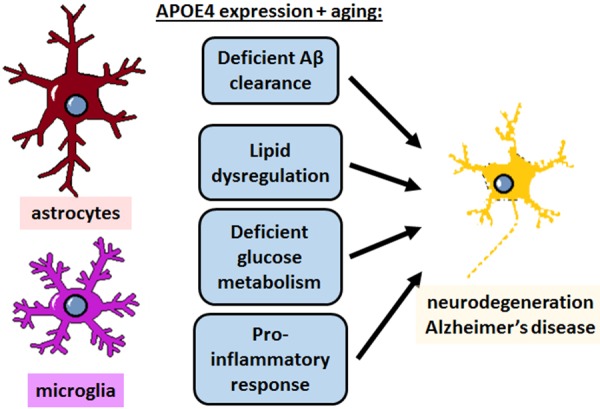
APOE4 disrupts homeostatic pathways in astrocytes and microglia to cause neurodegeneration and AD. APOE4 expression and the normal aging process itself impair astrocyte and microglia physiology in specific pathways, which could theoretically be targeted to treat AD. In addition to deficient clearance of Aβ, emerging evidence specifically highlights lipid dysregulation and deficient glucose metabolism in astrocytes, and a neurodegenerative pro-inflammatory response in microglia, and to some extent in astrocytes as well. All of these pathways converge with similar deficits that occur in normal aging to ultimately lead to neurodegeneration.

The role of APOE4 in mediating AD risk is complex and multifactorial, involving a diverse array of cell types and functions that need to be taken into consideration for APOE-directed drug development. Studies from the last decade have made significant progress in defining what those functions are, and how aging might factor into the progression of APOE4-mediated AD. Cholesterol metabolism, LD formation and lipid transfer from neurons to glia, and glucose/glycogen/lactate metabolism from glia to neurons all appear to be important pathways in maintaining brain health, particularly during aging; and the pro-inflammatory nature of APOE4 and decreased phagocytic capacity of APOE4-expressing glia likely contributes to neurodegeneration as well (Figure 6). These pathways may yield viable therapeutic targets for treating AD, but the precise mechanisms and connections with APOE4 still remain poorly defined. It is also unclear how APOE4-mediated disrupted function in astrocytes and microglia separately could synergize to increase AD risk, warranting further investigation.

## Author Contributions

CF wrote the first draft of the manuscript. CF, MH and WR wrote sections of the manuscript. MM and CF created the figures. All authors contributed intellectually and to manuscript revision, and read and approved the submitted version.

## Conflict of Interest Statement

The authors declare that the research was conducted in the absence of any commercial or financial relationships that could be construed as a potential conflict of interest.
